# Silicosis: I.—Administrative and Clinical

**Published:** 1931

**Authors:** J. A. Nixon

**Affiliations:** Professor of Medicine in the University of Bristol; Physician to the Bristol Royal Infirmary; Consulting Physician to Southmead Hospital


### SILICOSIS.

### I.—ADMINISTRATIVE AND CLINICAL.

BY

J. A. NIXON, C.M.G., M.D., F.R.C.P.,

*Professor of Medicine in the University of Bristol;*
*Physician to the Bristol Royal Infirmary;*
*Consulting Physician to Southmead Hospital.*

---

THE importance of recognizing chronic interstitial pneumonia or pulmonary fibrosis has increased of late because some forms are occupational in origin and may give rise to claims for compensation. Special medical schemes have been set up under the Workmen's Compensation Acts to deal with the compensation of workmen in certain industries.

Fibrosis of the lung may be classified as (*a*) pure fibrosis, *i.e.* no tuberculosis; (*b*) tuberculo-fibroid, *i.e.* tuberculosis becoming fibroid; (*c*) fibro-tuberculous, *i.e.* fibroid becoming tuberculous.

The distribution of the fibrosis may be local or diffuse.

1. *Local.*—This occurs in tuberculosis neoplasms, aneurysms, infarcts, compression of bronchi.

2. *Diffuse.*—This occurs in tuberculosis (but is apt to be unilateral), as a sequel of massive type of lobar pneumonia, after broncho-pneumonia (especially in measles, whooping-cough and influenza). In any type of recurrent bronchitis or broncho-pneumonia; in cases of asthma; and as result of inhalation of irritant gases. It may be due to syphilis, to thickened pleura, or lastly to pneumoconiosis.

*Pneumoconiosis* (Greek κονίω = I cover with dust).—This name implies that the fibrosis is due to dust-inhalation. Many dusts can irritate the lungs; but dusts composed largely of organic material require—apart from infections they may convey—long periods of time (twenty to thirty years) before they produce any demonstrable changes in the lungs. Dust heavily laden with silica ($SiO_2$) may produce extensive changes within two or three years (particularly if combined with an alkali; clay and coal-dust seem to retard the action of silica). It may be said that the danger of any dust is in direct proportion to the percentage of its silica content. We may, therefore, devote our time to the consideration of silicosis.

### INDUSTRIES IN WHICH SILICOSIS OCCURS.

1. Refractories industries, *i.e.* manufacture of ganister and silica bricks for metallurgical and other high temperature furnaces.

2. Sandstone industries, quarrying and dressing sandstone.

3. Granite industries, quarrying and dressing granite.

4. Slate industries, quarrying and dressing slate.

5. Metal-grinding, where steel instruments are ground on sandstone wheel, especially in "facing" a new grindstone before use.

6. Sand-blasting of metals.

7. Pottery manufacture. Ground flint is used to embed the articles of ware whilst they are being fired.

8. Silica crushing industries in which ground silica is used.

9. Tin mining, drilling silicious rock.

10. Coal mining, cutting through silicious stone to reach coal, and "dusting."

11. Asbestos industry; asbestos is magnesium silicate.

In these industries specialist medical boards have been appointed to make periodic examinations of the workmen both at entry and afterwards, also to examine for the issue of compensation certificates. The approach to these boards by individual claimants for compensation is via the regional medical officer. If he gives a certificate the claimant is examined by the board after the deposit of 30s.; if the regional medical officer refuses a certificate the claimant may still appeal to the board on depositing £8, of which £6 10s. is returned if the appeal succeeds.

SYMPTOMATOLOGY.—Silica dust may be invisible and is usually non-irritating, so that workers are unaware of the danger of inhalation.

*Chronic form.*—The first symptom is dyspnœa after many years' exposure in most cases, and it appears at first only on exertion. Apart from dyspnœa the symptoms are those of any respiratory disease, and depend on the character and degree of secondary infections and consequent changes. A French writer* has said: "The diagnosis of pulmonary silicosis is very difficult. It rests above all on the etiological data and on certain radiographic signs which are far from clear and require to be determined. In fact, in subjects presenting the signs of pulmonary sclerosis one thinks of silicosis when they have worked for some time in an industry where silica is present." In advanced but uncomplicated cases of general fibrosis of the lungs

* Policard, Magnin and Martin, *La Presse Medicale*, 1930, xxxviii. 875.

there may be a striking contrast between the extensive pulmonary lesions and the absence of marked constitutional symptoms.

*Acute form.*—A few cases after only two and a half to four years' exposure have manifested an acute form of silicosis. They first complained of dyspnœa. Then general constitutional disturbance developed with pyrexia, and an acute illness ended fatally in a few weeks. In these cases cyanosis was pronounced, and the whole picture resembled miliary tuberculosis.

### DIAGNOSIS OF SILICOSIS FROM OTHER CHRONIC DISEASES WITH FIBROSIS.

The symptoms and course as stated by the patient and relatives are most important. Particular attention must be paid to histories of previous pulmonary ailments like asthma, bronchitis, influenza, pneumonia, tuberculosis and exposure to irritant gases. The early symptoms are :—

Dyspnœa on exertion.

Cough, usually short and paroxysmal, occurring at night and on rising in the morning. As a rule, a little viscid sputum like an oyster is eventually expectorated.

Cyanosis, quite slight as a rule, but a valuable sign if present.

Hæmoptysis, but this generally indicates a tuberculosis infection.

Pleuritic pains are common and appear early.

Clubbed fingers are rare, and suggest bronchiectasis, bronchiolectasis or tuberculosis.

### PHYSICAL SIGNS.

Loss of weight is the rule, but it is not always rapid ; the general nutrition is below normal and the muscles are poor in bulk and tone. The chest expansion is diminished, but the measuring tape is unreliable. The vital capacity should be measured by means of a spirometer. In shape the chest is flat in front and round-shouldered behind. The infra-clavicular fossæ are hollowed, and the intercostal spaces are indrawn; there is sometimes marked recession on inspiration. Above the clavicles the apices of the lungs may appear to be drawn down in inspiration, not flat and fixed as in tuberculosis, because there are not the apical pleural adhesions of tuberculosis. The accessory muscles of respiration are in action. The skin is pale, and subcutaneous venules appear on the chest.

Precordial pulsation is well marked, because retraction of the edges of the lung leaves the heart uncovered.

Tactile fremitus is, as a rule, increased.

*Percussion.*—As the changes are bilateral no contrasts in resonance are obtained. The note is higher pitched than normal, and the superficial area of cardiac dullness is increased by "uncovering."

The apices give a more resonant note than the lower areas, and the area of root impairment is enlarged, but differing from old hilar tuberculosis, in that there is impairment above and below the root dullness.

*Auscultation.*—Poor air entry is the rule. The respiratory murmur is weakened, especially on the right side and at the base. It may have a blowing character over the upper part of the chest which gradually spreads to the whole lungs.

There may be a few fine rales and clicks in the root areas, in the axillæ and bases, but the presence of rhoncus, sibilus and mucous rales is not characteristic of silicosis depending rather on associated bronchitis or bronchiectasis.

In the right mammary region about the fourth space the breathing is often puerile or almost bronchial. Dry pleural friction is frequently heard and felt in advanced cases, usually in the right axillary line to begin with. Vocal resonance is increased particularly towards the apices, where whispering pectoriloquy may also be well heard.

This description applies only to cases uncomplicated by tuberculosis or bronchiectasis. The presence of these complications will introduce the innumerable physical signs by which they betray themselves.

The most important aid in the diagnosis of the fibrosis of the lung is, however, radiography, with which subject Dr. Bush has kindly undertaken to deal.

